# Editorial: Inguinal Hernia Emergency

**DOI:** 10.3389/jaws.2023.12013

**Published:** 2023-09-19

**Authors:** Pilar Hernández-Granados

**Affiliations:** General Surgery/Abdominal Wall Surgery, Fundación Alcorcón University Hospital, Rey Juan Carlos University, Alcorcón, Spain

**Keywords:** emergency inguinal hernia repair, laparoscopic emergency inguinal hernia repair, femoral hernia, elderly patients, open posterior approach

Elective inguinal hernia repair is one of the most frequent surgical procedures performed all over the world. Between 6% and 9% of these procedures are performed as emergency surgery, due to incarceration, strangulation or intestinal obstruction. Emergency inguinal hernia surgery has much higher mortality and morbidity rates than elective repair. Moreover, up to 15% of emergency inguinal hernia repairs need an intestinal resection, increasing the morbidity and mortality even more. Patients in emergency surgery tend to be older and have more comorbidities, as it has been described in the literature. Furthermore, this emergency surgery is performed worldwide in every kind of setting, small facilities in remote places or tertiary hospitals in big cities. And we cannot forget that urgent inguinal hernia is a life-threatening situation, and we will need to prioritize to save patient’s life above repair. Due to all these facts, there were controversies about which can be the best surgical technique to perform in emergency setting.

As long as there is no consensus about the recommended approach of these patients, this Special Issue gather the different techniques and approaches that can be done in emergency surgery. Open approach is the most common surgical technique used in emergency setting, in the majority of cases via an anterior approach (Lichtenstein repair, for example). Open posterior approach is less used and partially unknown by great number of surgeons, especially those non-dedicated to abdominal wall surgery. The article from Rodrigues-Gonçalves et al. compares the two approaches in a retrospective cohort, showed that open posterior preperitoneal mesh repair had better results in short and long terms than anterior approach in emergency setting. Perhaps we need to emphasize the teaching and learning of this kind of approach in daily basis in order to increase the use in emergency setting as well.

Laparoscopic repair in elective setting has been advocated as the gold-standard technique, although its use is very variable in different countries and facilities. The use of laparoscopic approach in emergency inguinal hernia repair is even more controversial. Moreno-Suero et al. show their experience and results comparing to open surgery and the conclusion is that laparoscopic approach is safe, feasible and effective, as far as there were experienced laparoscopic surgeons in the emergency setting.

First of all, the aim of emergency inguinal hernia surgery will be to reverse visceral ischemia, preventing the need of intestinal resection and avoiding sepsis, leading to decrease the morbidity of the procedure. Emphasizing this key point, Weitzner and Chen describe in their article the role of realising incisions in order to enlarge the defect and facilitate visceral reductions. This kind of incisions have been described in lectures and discussions between surgeons, but there is scarce literature describing their role in the practical management of emergency hernia surgery. They offer recommendations on how to perform these realising incisions in open and minimally invasive surgery, laparoscopic or robotic.

As it is mentioned before, emergency inguinal hernia repair patients are older and have more co-morbidities and therefore, have worst results with more complications and higher mortality than younger patients. Piltcher-da-Silva et al. show in their paper a systematic review of the literature about emergency inguinal hernia repair in the elderly group, and the conclusions confirmed that emergency surgery in the elderly group carries an increased risk of morbidity and mortality. As the elective groin surgery is very safe in aged patients, they suggest that elective surgery must be offered to this population instead watchful waiting.

Femoral hernias have great impact in the emergency setting. Their special characteristics made these hernias more prone to strangulation, due to the rigid margins of the defect. Forty five percent of all femoral hernias are operated on emergency basis. Moreover, the need of intestinal resection is greater in this kind of hernias. To avoid the laparotomy and to improve the evaluation of the viability of the herniated bowel, perhaps the minimally invasive approach could be a very good option for surgical treatment in the emergency. Shuttleworth et al. perform a systematic review about the utility of minimally invasive approach in the emergency management of femoral hernias and they concluded that laparoscopic approach in emergency femoral hernia repair is feasible and can be done safely with good results, similar to open surgery, but there are no good quality evidence in this Special Issue.

Although this Special Issue does not cover all the aspects of emergency inguinal hernia surgery, it really does give an overview of multiple important topics. We hope it will be helpful to interested readers, specially to those who perform emergency surgery on their daily practice.

